# 17q25.3 copy number changes: association with neurodevelopmental disorders and cardiac malformation

**DOI:** 10.1186/s13039-023-00644-2

**Published:** 2023-07-10

**Authors:** Nikhil Shri Sahajpal, David H. F. Jeffrey, Barbara R. DuPont, Benjamin Hilton

**Affiliations:** Diagnostic Laboratories, Greenwood Genetic Center, Greenwood, SC 29646 USA

**Keywords:** Rare copy number variants, Genotype–phenotype correlation, 17q25 region, NHEJ, Neurodevelopmental disorders

## Abstract

Copy number variants (CNVs) have been identified as common genomic variants that play a significant role in inter-individual variability. Conversely, rare recurrent CNVs have been found to be causal for many disorders with well-established genotype–phenotype relationships. However, the phenotypic implications of rare non-recurrent CNVs remain poorly understood. Herein, we re-investigated 18,542 cases reported from chromosomal microarray at Greenwood Genetic Center from 2010 to 2022 and identified 15 cases with CNVs involving the 17q25.3 region. We report the detailed clinical features of these subjects, and compare with the cases reported in the literature to determine genotype–phenotype correlations for a subset of genes in this region. The CNVs in the 17q25.3 region were found to be rare events, with a prevalence of 0.08% (15/18542) observed in our cohort. The CNVs were dispersed across the entire 17q25.3 region with variable breakpoints and no smallest region of overlap. The subjects presented with a wide range of clinical features, with neurodevelopmental disorders (autism spectrum disorder, intellectual disability, developmental delay) being the most common features (80%), then expressive language disorder (33%), and finally cardiovascular malformations (26%). The association of CNVs involving the critical gene-rich region of 17q25.3 with neurodevelopmental disorders and cardiac malformation, implicates several genes as plausible drivers for these events.

**Supplementary Information:**

The online version contains supplementary material available at 10.1186/s13039-023-00644-2.

## Introduction

The high variability in the human genome can be accounted for by polymorphisms, including both single nucleotide polymorphisms (SNPs) and copy number variation (CNV) polymorphisms, defined as changes that occur in > 1% of the human population [1, 2]. In terms of total bases involved, CNVs appear to have a higher contribution toward human variability and evolution compared to SNVs [3]. Several mechanisms such as nonallelic homologous recombination (NAHR), nonhomologous end-joining (NHEJ), retrotransposition, fork stalling, and template switching have been described as mechanisms for occurrence of CNVs with or without complex rearrangements in the genome [4]. In addition to the polymorphic CNVs, rare recurrent and non-recurrent CNVs can cause complex disorders. A few well-described CNV events associated with disorders include, Williams-Beuren syndrome (7q11.2 deletion; OMIM* 194050), Prader-Willi and Angelman syndromes (15q11-q13 deletion, OMIM* 176270, 105830), and DiGeorge/Velocardiofacial syndrome (22q11.2 deletion; OMIM* 188400).

Notably, several disorders associated with CNVs have been described on chromosome 17, namely, 17p13.3 telomeric duplication syndrome (OMIM* 612576), 17p13.3 deletion syndrome including the Miller-Dieker syndrome (OMIM* 247200), Smith-Magenis syndrome (OMIM* 182290), Chromosome 17q12 deletion syndrome (OMIM* 614527), Chromosome 17q12 duplication syndrome (OMIM* 614526), and Chromosome 17q23.1–q23.2 deletion syndrome (OMIM* 613355). Interestingly, chromosome 17 is among the smallest of chromosomes, but is the fifth most gene-rich. Of the 4690 OMIM genes, 16.6% (282) reside on chromosome 17, of which 10.9% (31/282) are within the most distal sub-band on 17q (17q25.3). The genes in the 17q25.3 region are involved in a wide range of cellular processes: *ASPSCR1* (OMIM* 606236) has been associated with tumors; *SEPTIN9* (OMIM* 604061) and *RNF213* (OMIM* 613768) are causal/associated with constitutional disorders (hereditary neuralgic amyotrophy and susceptibility to moyamoya disease, respectively). However, the literature is lacking in the phenotypic effects of CNVs in the 17q25.3 region, with only few case studies published to date. Of these, Probst et al. [5] described a series of eight cases with non-overlapping CNVs, with ~ 60% of cases associated with cardiac malformation. Additionally, Lukusa et al. [6] reported a microduplication in a patient with dysmorphormic features, growth restriction, and developmental delay.

Importantly, occurrence of CNVs in this region appears to be rare, deemed from the limited cases reported in the literature, and given the absence of segmental duplications in this region are non-recurrent. Herein, we present 15 cases with CNVs in the 17q25.3 region and expand the phenotypic features associated with CNVs in this region to neurodevelopmental disorders [autism spectrum disorder (ASD), intellectual disability (ID), developmental delay (DD)], expressive language disorder, and cardiac malformations.

## Methods

### Human subjects

An internal audit at our institution was performed to identify cases in the cytogenetics laboratory that were subjected to chromosomal microarray (CMA) analysis over an eleven-year period (2010–2022). A total of 18,542 cases were identified, which were queried for CNVs in the 17q25.3 region. The original data were re-analyzed and cases were categorized as (a) with sole finding of CNV in 17q25.3 region and (b) with other clinically reportable CNVs in addition to a 17q25.3 CNV. The clinical charts were reviewed to investigate genotype–phenotype correlation. Parental studies were performed, when possible, to determine the inheritance pattern of the CNVs. The study was approved by the Institutional Review Board of Greenwood Genetic Center and the analysis was performed in accordance with the institutional guidelines for human research.

### Cytoscan HD microarray (CMA)

The genomic DNA (gDNA) for CMA was analyzed using the CytoScan HD assay following the manufacturer’s protocol (ThermoFisher Scientific, USA). The data were analyzed using the Chromosome Analysis Suite (ChAs 3.0) software, where the signal for the sample was compared with a reference set. Differences in signal between the sample and reference were expressed as a log2 ratio and represent relative intensity for each marker. A discrete copy number value was determined from the relative intensity data. CNVs were confirmed by quantitative polymerase chain reaction technology.

### Data analysis

The following databases were used to assess the clinical significance of genomic aberrations less than 5 Mb: the database of genomic variants (DGV), DatabasE of genomiC variation, and Phenotype in Humans using Ensembl Resources (DECIPHER), Online Mendelian Inheritance in Man (OMIM), and PubMed. The clinical indications of the subjects studied in this cohort were correlated with the CNV region/gene(s), with other case(s) in our cohort and/or case(s) published in the literature/databases.

## Results and discussion

### Patient summary

A total of 15 cases were identified that harbored a CNV in the 17q25.3 region. The demographics and clinical characteristics of the patients included a mean age at diagnosis of 7.5 ± 6.9 SD (range 4 months to 22 years), and 33% (5/15) female and 66% (10/15) male. A total of 54 genetic tests were performed in the diagnostic laboratories of GGC between these 15 patients (ranging from one to seven), including the CytoScan HD microarray which was performed for all 15. These 15 subjects were clinically diagnosed with: ASD, ID and/or DD (80%; 12/15); expressive language disorder (33%; 5/15); and cardiovascular malformations (26%; 4/15). As a single trait, DD was the most common clinical feature, with a penetrance of 46% (7/15), followed by expressive language disorder 33% (5/15). Further, a majority of patients also exhibited percentiles below average for physical measurements, such as weight, height, and head circumference (Additional file 1). The available phenotypes of the family members for all cases has been listed in Additional file 2.

### CNV findings

**Fig. 1:**
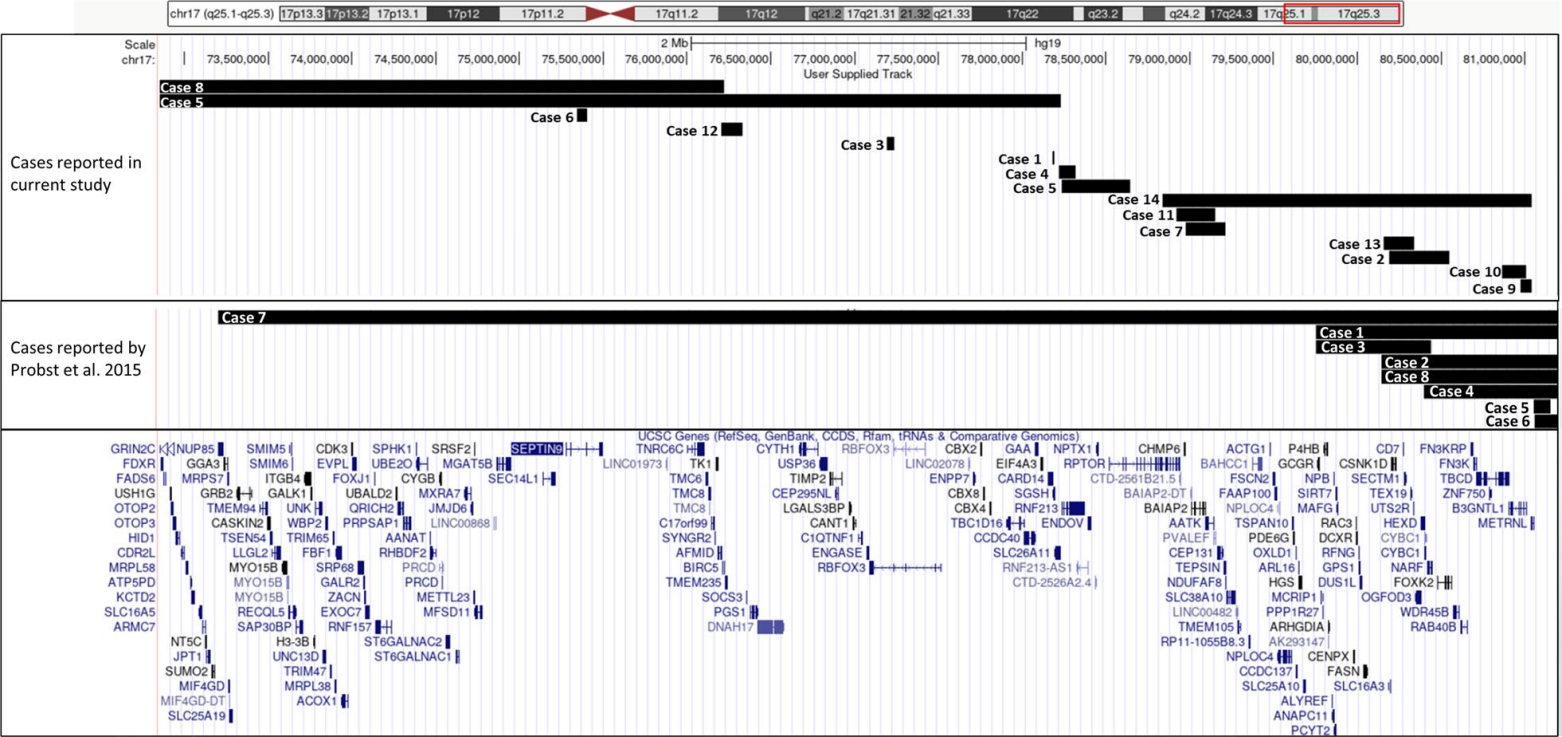
Depiction of the cases included in the study (case numbers) and CNVs overlapping the 17q25.3 region

Of the 15 cases, seven cases were found to have a CNV only in the 17q25.3 region with no other clinically reportable CNV (including variants of uncertain significance), while eight cases had CNVs in other regions in addition to the 17q25.3 region (Table 1). Of these seven cases, only one case had a pathogenic finding (trisomy 21), while the other cases had CNVs that were classified as variants of uncertain significance. The CNVs in the 17q25.3 region shared no “smallest region of overlap” or “breakpoint grouping” and were found to be of variable size and were dispersed throughout the region (Fig. 1). Notably, none of the CNVs extended to the chr17 telomere, indicating that these events were not likely to result from malsegregation of a translocation in the parents.

**Table 1:** Summary of CNV findings detected by CMA affecting candidate genes for neurodevelopmental disorders and cardiac malformation in 17q25.3

Case	Age	Sex	CMA	Size (kbp)	Gene(s)	Other tests results	Primary category (phenotype)
*Cases with isolated 17q25.3 region CNVs*							
1	5y	F	arr[GRCh37] 17q25.3(78183648_78190994) × 1	7	*SGSH*	Abnormal SGSH sequencing analysis and Heparan N sulfatase deficiency	NDD
2	23y	M	arr[GRCh37] 17q25.3(80190108_80552040) × 3 pat	362	*SLC16A3, CSNK1D, CD7, SECTM1, TEX19, UTS2R, OGFOD3, HEXD, CYBC1, NARF, FOXK2*	Normal karyotype and FMR1 test	NDD
3	1.9y	F	arr[GRCh37] 17q25.3(77190712_77234523) × 1	44	*RBFOX3*	Normal karyotype and FMR1 test	NDD
4	6y	M	arr[GRCh37] 17q25.3(78218807_78317442) × 3 pat	99	*SLC26A11* and *RNF213*	Normal karyotype and FMR1 test	EL, CM
5	5 m	M	arr[GRCh37] 17q25.1q25.3(72852763_78232958) × 3 ~ 4 dn, 17q25.3(78239107_78643088) × 1 dn	5380	Several including *SEPTIN9* and *EXOC7* (17q25.1)	Normal, HRAS, GCDH gene sequencing and normal biochemical profile	CM, NDD
6	1 m	F	arr[GRCh37] 17q25.3(75346425_75403493) × 3	57	*SEPTIN9*		NDD
7	9y	M	arr[GRCh37] 17q25.3(78976594_79215867) × 3	239	*BAIAP2, MIR657, MIR338, AATK, PVALEF, CEP131, TEPSIN, NDUFAF8*	Normal Organic acids	NDD
*Cases with other clinically reportable CNVs in addition of 17q25.3 region*							
8	5.3y	M	arr[GRCh37] 2q11.2(98448884_98811103) × 3 mat, 15q11.2(22770421_23620154) × 3 pat, 17q25.1q25.3(72832054_76221428) × 3 dn	3389	Several including *SEPTIN9* and *EXOC7* (17q25.1)	47,XY, + mar[16]/46,XY[14], FISH determined the marker to be chr17 material	EL, NDD
9	3.3y	F	arr[GRCh37] 17q25.3(80976346_81041938) × 3 mat, 19q13.41(52940574_53158834) × 3 mat	66	*B3GNTL1* and *METRNL*	Normal WES	EL, NDD
10	9y	F	arr[GRCh37] 6q24.1(141957503_142116632) × 1 mat, 17q25.3(80864260_81007175) × 3 mat	143	*TBCD* and *B3GNTL1*	Negative WES and normal biochemical profile	NDD
11	6y	M	arr[GRCh37] 8q13.2(69000406_69314786) × 3, 17q25.3(78922808_79,149,972) × 3	227	*RPTOR, CHMP6, BAIAP2, MIR657, MIR338, AATK, PVALEF*	Normal FMR1 testing	EL, NDD
12	20.6y	M	arr[GRCh37] 5p15.33(1708093_1816055) × 3, 17q25.3(76203763_76330192) × 3	126	*AFMID, BIRC5, TMEM235*	Normal for *NSD1* gene analysis, Prader willi/Angelman syndrome, and FMR1 test	EL, NDD
13	11y	M	arr[GRCh37] Yq11.221q11.23(19574920_28799937) × 0, 17q25.3(80159158_80340511) × 3	181	*CCDC57, SLC16A3, CSNK1D, CD7, SECTM1, TEX19, UTS2R*	Normal for *NSD1* gene analysis, Prader willi/Angelman syndrome test	NDD, CM
14	3y	M	arr[GRCh37] 5p15.33(914441_1247538) × 3, 17q25.3(78839225_81041938) × 4	2203	Several genes	Normal karyotype	NDD, CM
15	1y	M	arr[GRCh37] 17q25.3(80543137_80715054) × 4, 21q11.2q22.3(15006457_48097372) × 3	172	*FOXK2, WDR45B. RAB40B, FN3KRP, FN3K, TBCD*	Trisomy 21	Classic features of trisomy 21

*NDD* Neurodevelopmental disorder, *EL* Expressive language disorder, *CM* cardiac malformation

The CNVs in the 17q25.3 region were found to be rare events, with a prevalence of 0.08% (15/18542) observed in our cohort. Further, the CNVs were dispersed across the entire 17q25.3 region with variable breakpoints and no smallest region of overlap. The subjects were observed to have a wide range of clinical features, with neurodevelopmental disorders (ASD, ID, DD) being the most common feature (80%), followed by expressive language disorder (33%), and finally cardiovascular malformations (26%). Our results are largely in agreement with the study by Probst et al., with one major variation regarding penetrance, as Probst et al. [5] reported a higher penetrance of cardiac malformation (~ 60%). The difference in penetrance of this trait can be attributed to the variability in the CNV regions between cases in these studies, the genes involved, and the inheritance pattern of these CNVs. Herein, we have compiled the cases reported in literature along with the cases in our cohort and proposed genotype–phenotype correlations for a subset of the genes impacted by these CNVs. The detailed phenotype for each case is listed in Additional file 3. Of note, all CNVs involving 17q25.3 were classified as variants of uncertain clinical significance given the lack of supporting evidence from the medical literature, with the exception of one pathogenic variant, which encompasses an established haploinsufficient gene discussed herein. Since this is a retrospective study for which medical records were retrieved for phenotyping, the rates of clinical features should be considered as lower-bound estimates.

### Isolated CNVs involving 17q25.3

*Case 1* The subject was diagnosed with Sanfillipo syndrome type A and had a similarly affected father. Sequencing analysis of the gene *SGSH* identified a homozygous deletion of 11 nucleotides starting at position 1272 (c.1272_1282del11), which is predicted to result in a reading frame shift and ultimately an aberrant protein. Additionally, CMA analysis detected an approximately 7 kb deletion (arr[GRCh37] 17q25.3(78183648_78190994) × 1), resulting in loss of exons 2–8 in the *SGSH* gene. The CNV analysis clarified the results obtained by sequencing analysis, for which the homozygous 11 nucleotide deletion called by sequencing was the result of an 11 nucleotide deletion on one allele and deletion involving exons 2–8 on the other allele. Further, heparin-N-sulfatase (heparan sulfaminidase) was deficient, consistent with Sanfilippo syndrome type A. N-acetylglucosamine-6-sulfatase (type D) activity was found to be normal, suggesting against multiple sulfatase deficiency. Maternal testing was not be performed.

Overall, these results were consistent with a clinical diagnosis of Sanfillipo syndrome type A in this individual. Interestingly, only SNVs in the *SGSH* gene have been reported in association with Sanfilippo A, and to our knowledge, this is the first reported case of a deletion in the *SGSH* gene (no cases reported in ClinVar or published literature), that appears to be causal for Sanfillipo syndrome type A syndrome.

*Case 2* The subject was diagnosed with ASD, DD, low muscle tone, posterior cerebellar artery syndrome, and intellectual disability. At the time of exam, additional features included a receding hairline, a narrow nasal bridge, a deep philtrum, and flat feet arches. He has family history of a maternal first cousin with speech delay and his maternal half uncle and his daughter were diagnosed with ASD. The subject had a normal 46,XY constitution by karyotyping and showed no evidence of trinucleotide repeat amplification within the *FMR1* gene.

The CMA detected an approximately 362 kb duplication which was found to be paternally inherited (arr[GRCh37] 17q25.3(80190108_80552040) × 3 pat). The duplication included the genes *SLC16A3, CSNK1D, CD7, SECTM1, TEX19, UTS2R, OGFOD3, HEXD, CYBC1, NARF,* and *FOXK2* (partial). Importantly, two cases in the literature were identified with duplications involving the *FOXK2* gene identified in patient with similar clinical features. First, a patient with ID was reported with a 494 kb duplication (DICEPHER database), which also partially duplicated the *FOXK2* gene, as observed in our subject. Although the duplication identified in the current study is a partial duplication similar to the duplication reported in DICEPHER, it is uncertain whether *FOXK2* is disrupted. If these duplications are inverted it is possible *FOXK2* is disrupted leading to a loss-of-function; however, additional evidence is needed to confirm this. Second, a patient was reported with partial tetrasomy of 17q25.3, with a segment translocated to 10q disrupting the *FOXK2* gene [7]. Notably, depletion of Foxk transcription factors have been observed in association with genome-wide transcriptional mis-regulation and developmental arrest in zebrafish embryos [8].

*Case 3* The subject was diagnosed with ADHD, developmental disorder of scholastic skills, impulsiveness, poor social cues, difficulty processing emotions, delayed physical development, speech delay, and possible exposure to alcohol during pregnancy. Notable facial features include dysmorphic facial features, congenital deformities of the skull/face/jaw, and café au lait spot on inner buttock. The family history was unknown for this subject. She had a normal 46,XX constitution by karyotyping and showed no evidence of trinucleotide repeat amplification within the *FMR1* gene.

The CMA detected an approximately 44 kb deletion (arr[GRCh37] 17q25.3(77190712_77234523) × 1), resulting in a single exon deletion of exon 4 within the gene *RBFOX3*. The *RBFOX3* gene has been predicted to be loss-of-function intolerant (pLI score of 1) and functional studies have shown an important role in brain development. Knockout of the *RBFOX3* gene in mice has shown reduced brain mass, abnormal hippocampal dentate gyrus, but normal total body mass, and increased susceptibility to seizures [9, 10]. Exon 3 deletion of *RBFOX3* has been reported in an individual with Rolandic epilepsy [11], while a de novo missense change c.620C > T(p.A207V) has been associated with neonatal seizures [12]. Additionally, a patient with developmental delay carrying a balanced translocation, t(9;17)(q12;q24), disrupting *RBFOX3* is also reported [13]. These cases suggest a key role in neurodevelopment for this gene. Of the limited cases reported in the literature, along with our case, there appears to be a varying neurodevelopmental phenotype that may or may not include seizures.

### CNVs involving RNF213

*Case 4* The subject was diagnosed with mixed receptive-expressive language disorder, selective mutism, anxiety disorder, coordination impairment, and a heart murmur. An evaluation for ASD was performed, but did not result in a diagnosis of ASD. He had growth issues from a young age, including short stature and poor weight gain. His physical appearance includes mild over folding of the left upper helix, a slightly high arched anterior palate, shortening of fifth fingers, minimal 3–2 toe syndactyly bilaterally, slightly dark and dysplastic of the fifth toenails, and several small café au lait spots. He was also noted to have additional points on the back of his teeth, a trait that is shared with his father. The subject had a normal karyotype of 46,XY, and showed no evidence of trinucleotide repeat amplification within the *FMR1* gene.

The CMA detected an approximately 99 kb duplication that was paternally inherited (arr[GRCh37] 17q25.3(80190108_80552040) × 3 pat). The duplication partially duplicated both the *SLC26A11* and *RNF213* genes, of which *RNF213* (OMIM* 613768) is associated with autosomal dominant and recessive susceptibility to moyamoya disease-2. The clinical symptoms of moyamoya disease vary widely and include, but are not limited to, headaches, recurrent transient ischemic attacks, epileptic seizures, or disturbances of speech and cognition, developmental delays or disability, and cardiovascular malformations. The literature has demonstrated SNVs in *RNF213* are strongly associated with moyamoya disease, but no report of a CNV in this region has been associated with the disease. Receptive-expressive language disorder, coordination impairment, and developmental delays observed in this patient overlap with the clinical features of moyamoya disease. Importantly, heterozygotes have been reported to have a milder phenotype compared to homozygotes, with the inheritance pattern being both AR and AD [14–17]. Furthermore, knockout of *SLC26A11* in mice have demonstrated impaired motor performance [18]. The presence of a cytogenetic aberration in an unaffected parent may be considered evidence that the change is not pathogenic; however, many genetic conditions exhibit variable expressivity, where the same abnormality may cause disease in some individuals and not in others.

*Case 5* The subject was diagnosed with a mild left pulmonic stenosis, a 2/6 systolic murmur, bruxism, and central apnea. He has a medical history of feeding through a J-tube, gastroesophageal reflux, allergic rhinitis, periodic fevers, and newborn jaundice. He has behavioral concerns of screaming fits and sensitivity to loud noises. Notable physical features include a full nasal tip, slightly uplifted earlobes, macrocephaly, syndactyly of the third and fourth fingers on the left hand, a large anterior fontanelle, and primary upper extremity hypertonia. The subject’s sample was sequenced for the first coding exon of the *HRAS* gene and the entire *GCHD* gene and showed a normal result. Further, several biochemical markers that included, oligosaccharides, mucopolysaccharides, lysosomal activity, and organic acids were found to be normal with slightly elevated urine glutarylcarnitine levels.

**Fig. 2:**
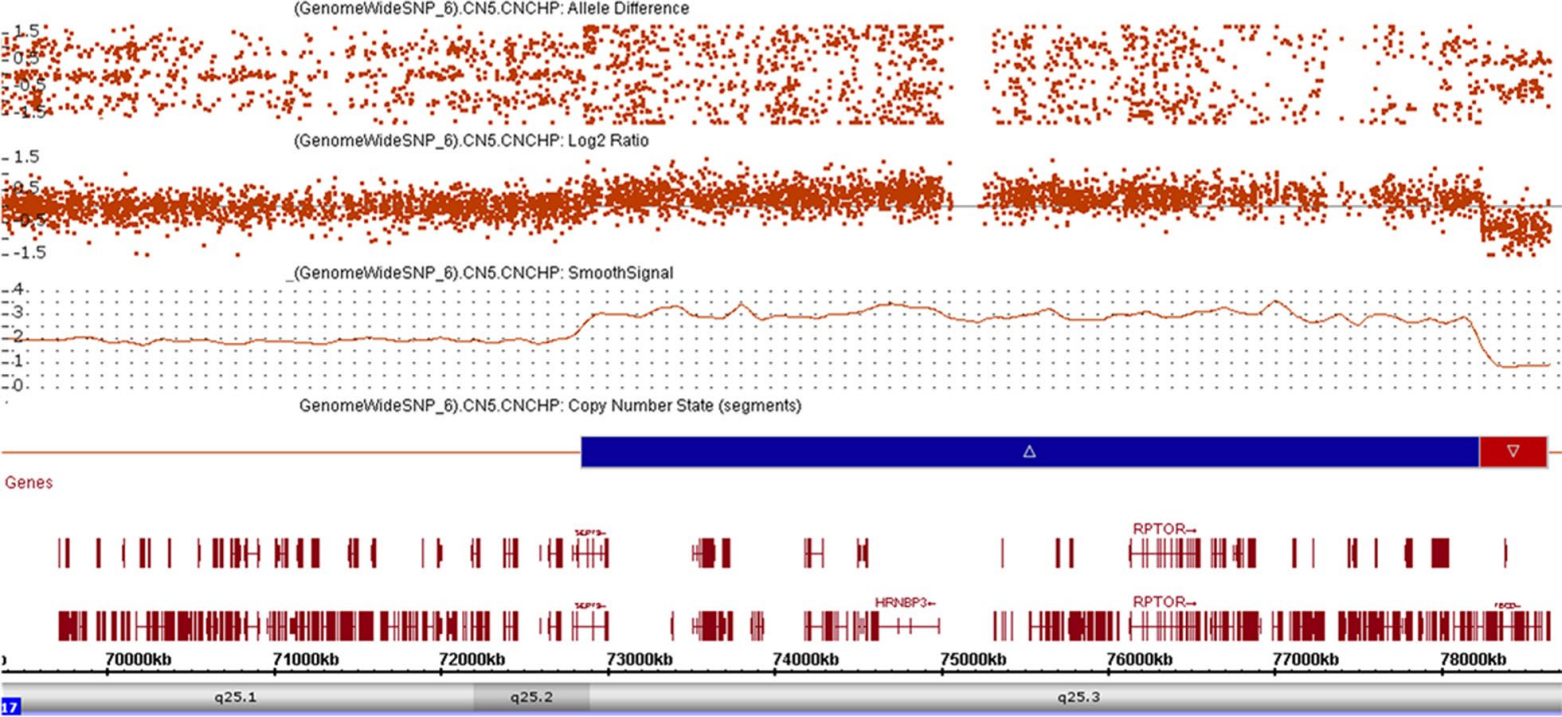
CMA showing the two copy number changes in the 17q25.2q25.3 region, arr[GRCh37] 17q25.2q25.3(72852763_78232958) × 3 ~ 4 dn, 17q25.3(78239107_78643088) × 1 dn, approximately 5380 kb and 404 kb in size, respectively

The CMA detected two de novo CNVs including an approximately 5.4 Mb 3–4 copy gain and a 404 kb deletion (arr[GRCh37] 17q25.1q25.3(72852763_78232958) × 3 ~ 4 dn,17q25.3(78239107_78643088) × 1 dn). The triplication encompassed several genes including *SEPTIN9* and *EXOC7*, while the deletion encompassed the genes *RNF213, ENDOV, NPTX1, RPTOR,* and *MIR4730* (Fig. 2). The gene *SEPTIN9* is associated with autosomal dominant hereditary neuralgic amyotrophy (OMIM* 604061) and *RNF213* (OMIM* 613768) is associated with moyamoya disease 2. Further, *EXOC7* at 17q25.1 has been associated with an autosomal recessive neurodevelopmental disorder with seizures and brain atrophy*,* while *RPTOR* has been reported to be intolerant to loss-of-function (pLI = 1). Functional studies have demonstrated that the loss of *RPTOR* in mice neural progenitor cells affects normal development in young age and may contribute to alleviate KA seizure‐induced behavioral abnormalities, suggesting that raptor protein plays an important role in seizure comorbidities [19]. Given that several genes are contributing to the phenotype observed in this case, it not possible to make a genotype–phenotype correlation exclusive to the genes in the 17q25.3 region. As an apparently de novo CNV, there is an increased likelihood that the 17q25.1q25.3 duplication and the 17q25.3 deletion contribute at least in part to the phenotypes observed in this patient.

### CNVs involving SEPTIN9

**Fig. 3:**
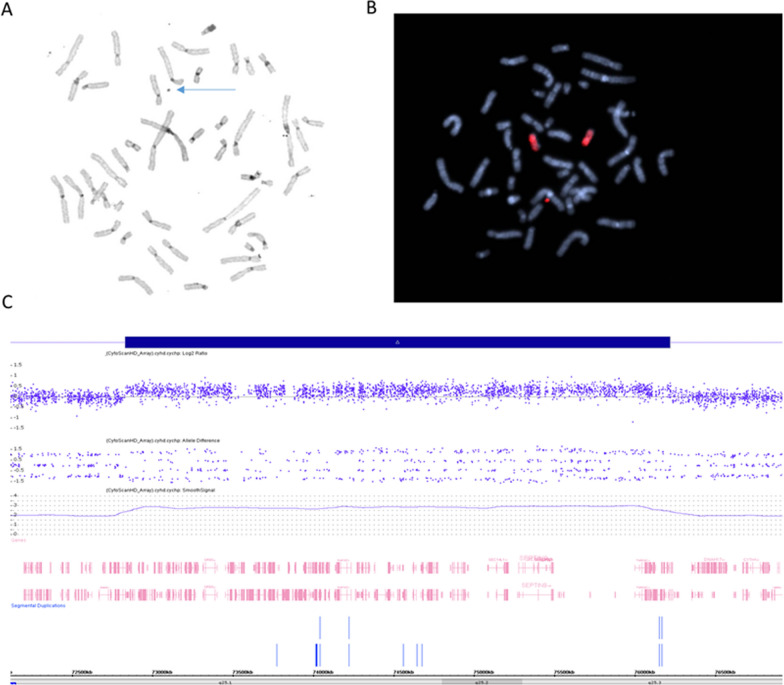
**A** Karyotype with the arrow showing the marker chromosome. **B** Metaphase FISH showing two chromosome 17 and the marker to be chromosome 17 material. **C** CMA showing the chromosome 17 material to be arr[GRCh37] 17q25.1q25.3(72832054_76221428) × 3, approximately 3389 kb in size

In addition to case 5, two other cases (cases 6 and 8) were identified with a duplication overlapping the *SEPTIN9* gene. *Case 8* The subject was diagnosed with expressive language disorder, learning disability, dyslexia, dolichocephalic, mild scoliosis, pes planus (flat foot), and reactive airway disease. He has a medical history of attention issues, speech delay, umbilical hernia, undescended testicle, and newborn jaundice. Notable physical features include ear anomalies (low set, posteriorly rotated, folded helix), high arched and narrow palate, long face, and down slated palpebral fissures, and below average physical measurements. Family history includes learning disability in the maternal uncle and below average physical measurements for the paternal great-uncle. The diagnostic testing for this subject included, karyotyping that showed 47,XY, + mar[16]/46,XY[14] and FISH that confirmed the marker as chromosome 17 material (Fig. 3a, b).

Follow-up CMA detected three CNVs: a maternally inherited 362 kb duplication within 2q11.2; a paternally inherited 850 kb duplication within 15q11.2; and a de novo approximately 3.4 Mb duplication within 17q25.1q25.3 (arr[GRCh37] 2q11.2(98448884_98811103) × 3 mat,15q11.2(22770421_23620154) × 3 pat,17q25.1q25.3(72832054_76221428) × 3 dn). The third CNV defines the region on chromosome 17 that is included on the marker chromosome (Fig. 3c). Of note, the large duplication on 17q contained *SEPTIN9*, that may account for the physical features and most likely the speech delay, expressive language disorder (focal paresis) observed in this patient, while the *EXOC7* duplication (and associated overexpression) may account for the learning disability. Missense variants in *EXOC7* have been previously reported in individuals with neurodevelopmental disorders [20, 21]. Given that the parents of this patient were unaffected, there is an increased likelihood that the 17q25.1q25.3 duplication contributes at least in part to the phenotypes observed in this patient.

*Case 6* The subject had a suspected diagnosis of Stickler syndrome and was found to have an approximately 57 kb duplication involving a single exon of *SEPTIN9* (arr[GRCh37] 17q25.3(75346425_75403493) × 3). Mortan et al., has previously reported a c.668-6582_668-6574delTGCCGCAGG in a patient with congenital heart disease [22] and missense variants have been associated with ASD and neuralgic amyotrophy [23, 24]. Notably, 14 cases with a variant impacting the coding region of *SEPTIN9* have been reported in the HGMD database, of which 10 were noted to have neuralgic amyotrophy.

### CNVs involving B3GNTL1

*Case 9* The subject was diagnosed with ASD, ID, and expressive language disorder. She has a medical history of DD, speech delay, and was born premature at 27 weeks. Notable physical features include a café au lait spot. She has family history of a maternal and a paternal cousin with ASD. She had a normal 46,XX constitution on evaluation with karyotyping and normal whole exome sequencing results. The CMA detected two CNVs, a 66 kb maternally inherited 17q25.3 duplication and a 218 kb maternally inherited 19q13.41 duplication (arr[GRCh37] 17q25.3(80976346_81041938) × 3 mat,19q13.41(52940574_53158834) × 3 mat). The duplication on 17q included the *B3GNTL1* and *METRNL* genes.

*Case 10* The subject was diagnosed with cerebral palsy, hypertonia, and spasticity. He has a medical history of delayed development, delayed milestones, and muscle spasms. He also has behavioral concerns of OCD-like behaviors. Notable physical features include an upturned nasal tip, café au lait spot on lower anterior neck, the fifth fingers are shortened bilaterally, and progressively dystonic features. The subject’s previous testing was normal for organic acids, total and free creatinine, amino acids, and acylcarnitine profile. The exome sequencing analysis detected a heterozygous missense variant (ADARc.3019G > A) that was found to be associated with dyschromatosis symmetrica hereditaria, but did not explain the observed phenotype. The CMA detected two CNVs, a 159 kb maternally inherited 6q24.1 deletion and a 143 kb maternally inherited 17q25.3 duplication (arr[GRCh37] 6q24.1(141957503_142116632) × 1 mat,17q25.3(80864260_81007175) × 3 mat). The duplication of 17q region contained the *TBCD* and *B3GNTL1* genes.

Interestingly, Probst et al*.* reported two cases of focal variants including the *TBCD* and *B3GNTL1* genes (one case each with loss or gain), neither of which included the more distal *METRNL* gene. Both reported cases were reported in association with DD and no cardiac malformation, as observed in our cases. Further, all three cases with duplication of the *TBCD* and *B3GNTL1* genes show facial dysmorphology. Thus, variants involving these two genes appear to be associated with DD, facial dysmorphisms, and no cardiac malformation. The two cases reported in literature were de novo, while both of our cases had CNVs that were maternally inherited. The mother was unaffected in both cases, supporting incomplete penetrance and variable expressivity for this variant.

### Limitations of the study

Though our study presents a set of cases with rare CNVs involving the 17q25.3 region, no smallest region of overlap could be established, which makes it difficult to ascertain definitive genotype–phenotype associations. However, since the incidence of CNVs involving 17q25.3 are rare, even in the disease population, and given that this is a gene-rich region of the genome, documentation of these cases is extremely important and may lead to definitive genotype–phenotype associations in the future. Importantly, cases 1–7 are the most informative, as these do not harbor any additional CNVs (and in some cases no sequencing alteration) suggesting a causal role for these CNVs/genes. Further, though the remaining cases (8–15) have additional CNVs, which complicates genotype–phenotype associations, all are of uncertain significance, and encourage further analysis of CNVs in the 17q25.3 region, given the rarity and a broader phenotype association of genes in this region. All efforts were made to review and provide detailed clinical information; however, all relevant phenotype information may not have been noted in all cases submitted for clinical genetic testing. Finally, evaluation of the disease mechanism is beyond the scope of this work, but these gene associations warrant further investigation in the future.

## Conclusion

Our study provides an in-depth analysis of a cohort of rare cases with CNVs involving the 17q25.3 region, that are dispersed across the entire 17q25.3 region with variable breakpoints and no smallest region of overlap. Importantly, we identified candidate genes including: *FOXK2* (pLI = 0.03) and *RBFOX3* (pLI = 1) associated with neurodevelopmental disorders; *TBCD* associated with dysmorphic facial features; and *RNF213* (pLI = 0)*, SEPTIN9* (pLI = 1)*,* and *EXOC7* (pLI = 0) associated with neurodevelopmental disorders and cardiac malformations (Table 2). However, genotype–phenotype correlation in additional cases with focal CNVs and/or pathogenic SNVs in these genes remains necessary to support our findings and ultimately establish causality. In summary, CNVs involving this region on 17q25.3 are associated with neurodevelopmental disorders (ASD, ID, DD), expressive language disorder, and cardiac malformations.

**Table 2:** Candidate genes identified for phenotype association

Case no. (s)	Literature case(s)	Candidate gene	Gene function and association	Phenotype implicated
2	DECIPHER (253687), One case reported by Hackmann et al. (reference no. [7])	*FOXK2*	*FOXK2* encodes a protein belonging to the conserved family of forkhead transcription factors that control a wide range of cellular processes, including the cell cycle and development (OMIM* 147685)	NDD
3	[10–12]	*RBFOX3*	*RBFOX3* encodes a member of the RNA-binding FOX protein family which is involved in the regulation of alternative splicing of pre-mRNA (OMIM* 616999)	NDD
4 and 5	OMIM* 613768	*RNF213*	*RNF213* encodes a protein that possesses both ubiquitin ligase activity and ATPase activity and is involved in protein–protein interaction. Associated with moyamoya disease 2, susceptibility to, AD, AR (OMIM* 613768)	NDD and CM
5, 6 and 8	[22–24]	*SEPTIN9*	*SEPTIN9* encodes a protein involved in cytokinesis and cell cycle control. Associated with autosomal dominant hereditary neuralgic amyotrophy (OMIM* 604061)	NDD and CM
5 and 8	[20, 21]	*EXOC7*	*EXOC7* encodes a protein that is a component of exocyst family. The exocyst complex plays a critical role in vesicular trafficking and the secretory pathway by targeting post-Golgi vesicles to the plasma membrane. Associated with neurodevelopmental disorder with autosomal recessive seizures and brain atrophy (OMIM* 608163)	NDD and CM
10	Two cases reported by Prost et al.	*TBCD*	*TBCD* encodes a protein that modulates microtubule dynamics by capturing GTP-bound TUBB. Associated with autosomal recessive encephalopathy, progressive, early-onset, with brain atrophy and thin corpus callosum (OMIM* 617193)	Dysmorphic facial features

*NDD* Neurodevelopmental disorder, *CM* Cardiovascular malformations

## Supplementary Information

**Additional file 1.** Physical measurements and percentiles for 15 cases harboring CNVs in the 17q25.3 region.

**Additional file 2.** Available phenotypes of family members for all 15 cases harboring CNVs in the 17q25.3 region.

**Additional file 3.** Clinical interpretation and associated clinical descriptions for 15 cases harboring CNVs in the 17q25.3 region.

## Data Availability

All data generated or analyzed during this study are included in this published article and its supplementary information files.

## Abbreviations

CNV: Copy number variant
SNV: Single nucleotide variant
ASD: Autism spectrum disorders
ID: Intellectual disability
DD: Developmental delay
AOH: Absence of heterozygosity
CMA: Chromosomal microarray
