# Supplementary material for: 17q25.3 copy number changes: association with neurodevelopmental disorders and cardiac malformation
Source: Mol Cytogenet. 2023 Jul 10;16:15. doi: 10.1186/s13039-023-00644-2 (PMC10334611; doi:10.1186/s13039-023-00644-2)
Supplement: Supplementary file 1 — Additional file 1. Physical measurements and percentiles for 15 cases harboring CNVs in the 17q25.3 region. [file 13039_2023_644_MOESM1_ESM.docx]

Supplementary file 1. Physical measurements and percentiles

| **Patient No.** | **Sample ID** | **Age** | **Weight** | | **Height** | | **Head Circumference** | |
| --- | --- | --- | --- | --- | --- | --- | --- | --- |
|  |  |  | **Physical** | **Percent** | **Physical** | **Percent** | **Physical** | **Percent** |
| 1 | 14-17744CMDX | 6yrs and 1 month | 16.8Kg | 3-10% | 106.7cm | 3% | 49.4cm | 3-10% |
| 2 | 16-14242CMDX | 6yrs and 4 months | 185Kg | 25% | 113.3cm | 25% | 50cm | 10% |
| 3 | 13-08614CMDX | 4.5 months | 5.95Kg | 7% | 55cm | >1% | 39cm | >3% |
|  |  | 14 months | 9.95Kg | 20% | 78cm | 45% | 43.5cm | 3% |
|  |  | 2yrs and 3 months | 12.8Kg | 40% | 83.8cm | 6% | 46cm | >3% |
|  |  | 3yrs and 6 months | 17Kg | 85% | 94cm | 15% | 47cm | 3% |
|  |  | 5 years | 24.5Kg | 95% | 104.5cm | 30% | 48.5cm | 3% |
| 4 | 18-06612CMDX | 22 years | 63.2Kg | NA | 174cm | NA | 56cm | 30% |
| 5 | 19-08391CMDX | 2 years | 15.2Kg | 95% | 88.3cm | 57% | 48.3cm | 53% |
|  |  | 3 years | 16.7Kg | 88% | 97cm | 64% | 49.5cm | 54% |
| 6 | 19-09135CMDX | 11 years | 28.8Kg | 5% | 137.2cm | 10% | 53.9cm | 69% |
| 7 | 12-09536CMCS | 5 years | 15.1Kg | 3% | 103.9 | 5-10% | 50.5cm | 25-50% |
|  |  | 7 years | 19.5Kg | 3-10% | 113.5cm | 3-10% | 51cm | 25% |
|  |  | 12 years | 41.5Kg | 39% | 153cm | 47% | 53cm | 24% |
| 8 | 13-01405CMCS | 20 years | 135Kg | >97% | 172cm | 25-50% | 59.8cm | 97% |
| 9 | 09-12073CMCS | 11 years | 85.2Kg | >99% | 151.1cm | 80% | 60cm | 99% |
|  |  | 15 years | 106Kg | >99% | 167cm | 10-25% | 62cm | 97% |
| 10 | 14-06241CMCS | 3 years | 13.15 | 25% | 91.4cm | 10-25% | 50cm | 50% |
|  |  | 8yrs and 8 months | 23.1Kg | 10-25% | 127.4cm | 10-25% | 52.8cm | 50-75% |
| 11 | 14-13677CMCS | 9 years | 23.5Kg | 11% | 122cm | 3% | 50cm | 5% |
|  |  | 10 years | 25Kg | 10% | 127cm | 10% | 51cm | 10% |
|  |  | 11.5 years | 30.6Kg | 10% | 134.5cm | 5% | 51.1cm | 3% |
| 12 | 15-08589CMCS | N/A | | | | | | |
| 13 | 19-13506CMCS | 21 months | 9.53Kg | <1% | 28.3cm | <1% | 47.6cm | 29% |
|  |  | 2 years | 10.8 | 2% | 78.7cm | <1% | 48cm | 7% |
| 14 | 13-02735CMCA | N/A | | | | | | |
| 15 | 11-15545CMHR | 4 months | 5.8Kg | 3-10% | 62.2cm | 10-25% | 42.4cm | 25-50% |
|  |  | 1 year | 8Kg | 3% | 71.5cm | 3-10% | 46.4cm | 75-90% |
|  |  | 2 years | 11.6Kg | 10-25% | 82cm | 3-10% | 49cm | 50-75% |
