# Supplementary material for: 17q25.3 copy number changes: association with neurodevelopmental disorders and cardiac malformation
Source: Mol Cytogenet. 2023 Jul 10;16:15. doi: 10.1186/s13039-023-00644-2 (PMC10334611; doi:10.1186/s13039-023-00644-2)
Supplement: Supplementary file 2 — Additional file 2. Available phenotypes of family members for all 15 cases harboring CNVs in the 17q25.3 region. [file 13039_2023_644_MOESM2_ESM.docx]

| **Case ID** | **Family History** |
| --- | --- |
| 1 | Father is Diagnosed with Sanfillipo Syndrome Type A |
| 2 | Maternal cousin history of speech delay  Maternal half-uncle and his daughter diagnosed with high functioning Autism |
| 3 | Unknown Family history due to impeding circumstances of being removed from the home due to neglect at a young age |
| 4 | Two maternal uncles with mental health issues, one with learning disabilities and one has a son with learning disabilities, Apraxia, and Dyslexia  Mother has a maternal cousin with Schizoaffective disorder whose son has Tourette syndrome  Maternal Aunt with Scoliosis  Father had additional point on tooth  Father has a paternal uncle with learning disabilities  Paternal grandmother had some siblings die in infancy of unknown cause |
| 5 | Parents had one prior miscarriage at three months  Paternal adult males’ history of Strokes at an early age  Paternal cousin has Hydrocephalus and developmental delays  Sister is diagnosed with Blue Sclera  Mother and maternal grandparents have history of migraines  Maternal grandmother has Bipolar Disorder |
| 6 | Not available |
| 7 | Mother diagnosed with Aneurysm, Irritable bowel syndrome and migraines  Father has symptoms of Asperger  Paternal uncle required special education  Maternal uncle has history of seizures  Sister with ADHD, Anxiety and Depression |
| 8 | Mother has Ulcerative Colitis  Family for maternal and paternal is average or below height  Half-sister has Anxiety and Asthma  Maternal uncle with learning disabilities  Maternal cousins with significant intellectual disorder and ADHD  Paternal great-uncle who has significant intellectual disability and hydrocephalus  Maternal grandfather has Coronary Artery disease and diabetes  Paternal grandparents both have Hypertension |
| 9 | Mother had two previous miscarriages, one which was a twin conception that spontaneously aborted at three months  Female maternal cousin has seizures  Male maternal cousin has concerning behavior that may be Autism  Maternal great aunt with learning problems and growth issues of unknown etiology  Paternal cousin born with leg length discrepancy  Female paternal cousin has Autism and possible case of Autism on father’s maternal family  Maternal and paternal grandparents have diabetes and Hypertension |
| 10 | Mother has a paternal cousin with Conduct Disorder |
| 11 | Both parents have history of learning problems  Mother has a maternal half-brother with learning disabilities |
| 12 | Maternal uncle with Down syndrome  Paternal uncle with Autism |
| 13 | Mother has history of alcohol and drug abuse  Mother also has multiple Sclerosis and is legally blind |
| 14 | Mother has history of pain pill addiction and is diagnosed with Hyperthyroidism  Maternal uncle had growth issues as a teenager  Maternal aunt that was stillborn and had IUGR  Maternal grandfather has history of Hypospadias  Maternal grandmother is diagnosed with Hypertension  Paternal grandmother died of Breast Cancer |
| 15 | Father and paternal grandparents have hearing loss  Paternal uncle with Spinal Bifida and another with congenital heart defect  Maternal aunt with Bi-Polar disorder, with a son that has ADHD |
